# Editorial: Targeting Bruton Tyrosine Kinase

**DOI:** 10.3389/fcell.2022.909655

**Published:** 2022-04-26

**Authors:** Cornelia Brunner, Annika C. Betzler, Jennifer R. Brown, Amy H. Andreotti, Emanuela Grassilli

**Affiliations:** ^1^ Department of Otorhinolaryngology, Head and Neck Surgery, Ulm University Medical Center, Ulm, Germany; ^2^ Chronic Lymphocytic Leukemia Center, Division of Medical Oncology, Dana-Farber Cancer Institute and Harvard Medical School, Boston, MA, United States; ^3^ Roy J. Carver Department of Biochemistry, Biophysics and Molecular Biology, Iowa State University, Ames, IA, United States; ^4^ Laboratory of Molecular Medicine, Department of Medicine and Surgery, University of Milano-Bicocca, Monza, Italy

**Keywords:** BTK, innate and adaptive immune response, inflammation, autoimmune diseases, B-cell neoplasia, solid tumors

The discovery of the Bruton tyrosine kinase (BTK) as the product of the defective gene in X-linked agammaglobulinemia (XLA) dates to almost 30 years ago (Tsukada et al., 1993). However, the more we study BTK, the more facets we discover in a protein once believed to be one size-fits-all and hematopoietic-specific. In this Research Topic, we present reviews and primary research providing insights into the complex role of BTK and an update on the latest findings regarding BTK as therapeutic target in inflammation, autoimmunity and malignancy.

Shortly after its discovery, BTK was positioned in the signal transduction pathway downstream of the B cell antigen receptor (BCR) and therefore relevant to adaptive immunity (Woyach et al., 2012). Upon BCR engagement, proteins including BTK, VAV, BLNK, PLC-γ2, and PI3K interact to form one signaling complex termed the signalosome (Seda and Mraz, 2015). Since BTK and VAV proteins are both components of the signalosome, Betzler et al. investigated whether these molecules lie in the same or independent pathways. Their data suggest a BTK-independent role for VAV1, but a BTK-dependent role for VAV3. Thus, these findings indicate that within the signalosome different VAV-family members can coordinate BCR-mediated signaling in different ways—necessary for BTK-dependent as well as independent signal transduction.

During the years it became evident that BTK is pivotal for transducing activation signals through receptors other than the BCR, such as CD19, BAFFR, TLRs, and chemokine receptors (Satterthwaite et al., 2000; Shinners et al., 2007; McDonald et al., 2021). In addition, BTK engagement in macrophages, myeloid cells, and mast cell activation revealed a role in the innate immune system. Recently, it was discovered that BTK regulates the NLRP3 inflammasome, involved in several acute and chronic disorders such as myocardial infarction, stroke, liver inflammation, type 2 diabetes, Alzheimer’s disease, Parkinson’s disease, and sepsis (Liu et al., 2017). The mini review by Weber addresses the role of BTK in NLRP3 inflammasome activation and highlights the resulting possibilities of targeting NLRP3-mediated inflammation using existing or future BTK inhibitors.

In the context of inflammatory and systemic autoimmune diseases, Neys et al. review the numerous studies supporting the potential of BTK targeting in conditions such as rheumatoid arthritis, systemic lupus erythematosus, multiple sclerosis, atherosclerosis, and many others.

The discovery of dysregulation of BTK expression or activation in pathologies characterized by excessive B cell proliferation/activation—e g., B cell neoplasias and auto-immune disorders—led to the development of several specific BTK inhibitors (BTKis), a few of which are already approved for some B cell malignancies. In addition, numerous others BTKis are currently in clinical trials. The review by Estupiñán et al. provides a comprehensive overview about the currently available BTKis. They present ongoing clinical trials and discuss the unique properties of the different BTKis and their possible limitations arising from resistance mutations. Furthermore, they point out possible adverse effects of the BTKis and discuss their underlying mechanisms.

An important issue when targeting a kinase is understanding the detailed molecular mechanisms that control regulation. So far, the strategy has been to block the kinase domain via irreversible or reversible occupation of the ATP binding site. However, this approach can lead to off-target effects given that the targeted Cys residue is conserved in a small number of other kinases. The identification and/or synthesis of allosteric inhibitors may therefore allow an ever-increasing and more stringent specificity of next generation BTKis. The “hot topic” of understanding the role of interdomain interactions and their importance in regulating BTK activity is reviewed by Kueffer et al.

In the last decade it has been discovered that BTK does not occur in only one flavor and, most notably, that other isoforms seem to be primarily expressed in solid tumors, rather than in immune cells. p65BTK and p80BTK (BTK-C) were most recently identified as oncogenic isoforms in multiple solid tumors (Eifert et al., 2013; Kokabee et al., 2015; Grassilli et al., 2016; Giordano et al., 2019; Sala et al., 2019; Wang et al., 2016; Wang et al., 2017). The expression of these BTK isoforms in solid tumors is associated with tumor progression and poor prognosis, suggesting the use of BTKis not only in the treatment of B-cell malignancies and autoimmune or inflammatory disease, but also in solid tumors.

Grassilli et al. review the potential of p65BTK—an oncogenic isoform identified and characterized in colon carcinoma—as a novel biomarker and highlight its potential as a therapeutic target in several solid tumors. To complete the picture, Wang et al. review the newly described oncogenic BTK isoforms and further focus on the BTK-C isoform and its therapeutic potential in epithelial tumors. Complementarily, the perspective article by Uckun and Venkatachalam underlines the potential of BTK as a molecular target for the treatment of B cell malignancies and solid tumors. Moreover, they discuss the role of BTK in the tumor microenvironment and the current knowledge of coumarins as possible BTKis.

In conclusion, this Research Topic summarizes and discusses how recent research has expanded our understanding of BTK activation in several different cell types and has led to the identification of novel isoforms. Notably, the understanding of BTK’s new roles, such as in inflammation and solid tumors, will create opportunities to re-purpose BTKis for treating an increasing number of diseases. In particular, experimental evidence supporting the addition of BTKis to bypass tumor resistance to chemo- and targeted therapy is very promising. To better repurpose the BTKis, an essential challenge is to define the biological functions of each isoform and identify the relevant signaling pathways in which they are involved. Finally, given the off-target effects of several BTKis - all targeting the kinase’s ATP-binding domain—and the onset of drug resistance, significant efforts to develop ever more specific inhibitors are quite valuable. It is anticipated that understanding the molecular mechanism(s) that regulate and fine-tune kinase activity will allow the development of allosteric inhibitors to overcome these problems, thus paving the way for BTKis to take center stage in the era of personalized medicine.

## References

[B1] Eifert C. Wang X. Kokabee L. Kourtidis A. Jain R. Gerdes M. J. (2013). A Novel Isoform of the B Cell Tyrosine Kinase BTK Protects Breast Cancer Cells from Apoptosis. Genes Chromosomes Cancer 52, 961–975. 10.1002/gcc.22091 23913792 PMC5006942

[B2] Giordano F. Vaira V. Cortinovis D. Bonomo S. Goedmakers J. Brena F. (2019). p65BTK Is a Novel Potential Actionable Target in KRAS-mutated/EGFR-Wild Type Lung Adenocarcinoma. J. Exp. Clin. Cancer Res. 38, 260. 10.1186/s13046-019-1199-7 31200752 PMC6570906

[B3] Grassilli E. Pisano F. Cialdella A. Bonomo S. Missaglia C. Cerrito M. G. (2016). A Novel Oncogenic BTK Isoform Is Overexpressed in colon Cancers and Required for RAS-Mediated Transformation. Oncogene 35, 4368–4378. 10.1038/onc.2015.5010.1038/onc.2015.504 26804170 PMC4994017

[B4] Kokabee L. Wang X. Sevinsky C. J. Wang W. L. W. Cheu L. Chittur S. V. (2015). Bruton's Tyrosine Kinase Is a Potential Therapeutic Target in Prostate Cancer. Cancer Biol. Ther. 16, 1604–1615. 10.1080/15384047.2015.1078023 26383180 PMC4846115

[B5] Liu X. Pichulik T. Wolz O.-O. Dang T.-M. Stutz A. Dillen C. (2017). Human Nacht, Lrr, and Pyd Domain-Containing Protein 3 (Nlrp3) Inflammasome Activity Is Regulated by and Potentially Targetable through Bruton Tyrosine Kinase. J. Allergy Clin. Immunol. 140, 1054–1067. e10. 10.1016/j.jaci.2017.01.017 28216434

[B6] McDonald C. Xanthopoulos C. Kostareli E. (2021). The Role of Bruton's Tyrosine Kinase in the Immune System and Disease. Immunology 164, 722–736. 10.1111/imm.13416 34534359 PMC8561098

[B7] Sala L. Cirillo G. Riva G. Romano G. Giussani C. Cialdella A. (2019). Specific Expression of a New Bruton Tyrosine Kinase Isoform (p65BTK) in the Glioblastoma Gemistocytic Histotype. Front. Mol. Neurosci. 12, 2. 10.3389/fnmol.2019.00002 30733667 PMC6353843

[B8] Satterthwaite A. B. Willis F. Kanchanastit P. Fruman D. Cantley L. C. Helgason C. D. (2000). A Sensitized Genetic System for the Analysis of Murine B Lymphocyte Signal Transduction Pathways Dependent on Bruton's Tyrosine Kinase. Proc. Natl. Acad. Sci. U.S.A. 97, 6687–6692. 10.1073/pnas.110146697 10829070 PMC18703

[B9] Seda V. Mraz M. (2015). B-cell Receptor Signalling and its Crosstalk with Other Pathways in normal and Malignant Cells. Eur. J. Haematol. 94, 193–205. 10.1111/ejh.12427 25080849

[B10] Shinners N. P. Carlesso G. Castro I. Hoek K. L. Corn R. A. Woodland R. L. (2007). Bruton's Tyrosine Kinase Mediates NF-Κb Activation and B Cell Survival by B Cell-Activating Factor Receptor of the TNF-R Family. J. Immunol. 179, 3872–3880. 10.4049/jimmunol.179.6.3872 17785824

[B11] Tsukada S. Saffran D. C. Rawlings D. J. Parolini O. Allen R. C. Klisak I. (1993). Deficient Expression of a B Cell Cytoplasmic Tyrosine Kinase in Human X-Linked Agammaglobulinemia. Cell 72, 279–290. 10.1016/0092-8674(93)90667-f 8425221

[B12] Wang J. D. Chen X. Y. Ji K. W. Tao F. (2016). Targeting Btk with Ibrutinib Inhibit Gastric Carcinoma Cells Growth. Am. J. Transl Res. 8, 3003–3012. Epub 2016/08/11. 27508020 PMC4969436

[B13] Wang J. Liu X. Hong Y. Wang S. Chen P. Gu A. (2017). Ibrutinib, a Bruton's Tyrosine Kinase Inhibitor, Exhibits Antitumoral Activity and Induces Autophagy in Glioblastoma. J. Exp. Clin. Cancer Res. 36, 96. 10.1186/s13046-017-0549-6 28716053 PMC5513110

[B14] Woyach J. A. Johnson A. J. Byrd J. C. (2012). The B-Cell Receptor Signaling Pathway as a Therapeutic Target in CLL. Blood 120, 1175–1184. 10.1182/blood-2012-02-362624 22715122 PMC3418714

